# Targeting the Tumor Microenvironment in Renal Cell Cancer Biology and Therapy

**DOI:** 10.3389/fonc.2019.00490

**Published:** 2019-06-14

**Authors:** Isabel Heidegger, Andreas Pircher, Renate Pichler

**Affiliations:** ^1^Department of Urology, Medical University of Innsbruck, Innsbruck, Austria; ^2^Department of Internal Medicine, Hematology and Oncology, Medical University of Innsbruck, Innsbruck, Austria

**Keywords:** tumor microenvironment (TME), renal cell cancer, therapy, anti-angiogenesis, immunotherapy, combination therapy

## Abstract

Renal cell cancer (RCC) is a highly vascularized and immunogenic tumor type. The inhibition of vessel formation by anti-angiogenic therapies, as well as the stimulation of the immune system by immunotherapy has revolutionized the therapeutic landscape of RCC in recent years. Nevertheless, both therapies are associated with therapy resistance due to a highly dynamic, adaptive and heterogeneous tumor microenvironment (TME). The aim of this short review article is to provide an overview of the components of the RCC TME as well as to discuss their contribution to disease progression. In addition, we report on preclinical and clinical findings and how the different TME components can be modulated to impede treatment progression as well as to overcome therapy resistance to anti-angiogenic or immunomodulating therapy concepts. Furthermore, we discuss the predictive and prognostic role of the TME in RCC therapy. We also report on the concept of combinational targeting of anti-angiogenic therapies and immune checkpoint inhibitor therapy, also including the latest results of clinical studies discussed at recent oncological meetings. Finally, promising new therapeutic targets within the TME are mentioned.

## Introduction

Renal cell carcinoma (RCC) is the seventh most frequently diagnosed malignancy, with a rising incidence in the developed world (1). The most common histological subtype is clear cell renal cell carcinoma (ccRCC), which is associated with a poor clinical outcome as up to 40% of ccRCC patients develop metastases, providing for a 5-year survival rate of 10% (2). Fortunately, in recent years improved knowledge of disease biology has led to significant efforts in the treatment of patients with advanced ccRCC. Consequently, targeted agents and immunotherapies have been introduced into daily routines.

**Figure 1:**
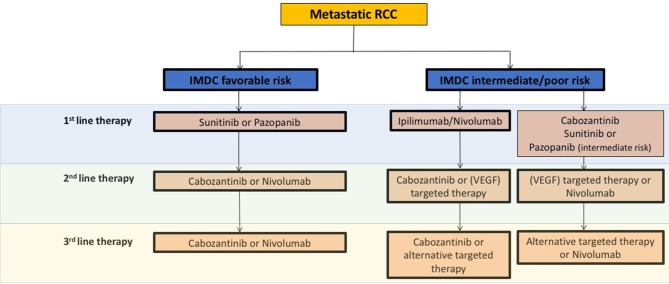
Schematic overview and recommendations from the current European Association of Urology 2018 guidelines for systemic treatment in mRCC. VEGF, vascular endothelial growth factor; IMDC, The International Metastatic Renal Cell Carcinoma Database Consortium.

Generally, ccRCC is a highly vascularized tumor, in which the von Hippel Lindau (VHL) tumor suppressor gene is frequently inactivated, leading to the overexpression of the hypoxia-inducible factor (HIF)-2α oncoprotein and its downstream targets like the vascular endothelial growth factor (VEGF) (3). Consequently, in recent years anti-angiogenic agents (AA) targeting the VEGF pathway like VEGF receptor (VEGFR) and tyrosine kinase inhibitors (TKIs) have been shown to improve disease control in large studies as well as in daily routines (Figure 1).

Besides AA therapies, immunotherapeutic strategies have evolved in the past years. For instance the programmed cell death 1 ligand-1 (PD-1) checkpoint inhibitor nivolumab was approved in AA pre-treated metastatic RCC (mRCC) patients based on the phase III CheckMate 025 study, which demonstrated overall survival (OS) and overall response rate (ORR) benefits as compared to the mTOR inhibitor everolimus (4) (Figure 1). Mechanistically, immune checkpoint inhibitors (ICB) specifically target immune checkpoint receptors or their ligands, thereby attacking mechanisms used by tumor cells to evade immune attack as well as restoring the ability of cytotoxic T cells to make an anti-tumor response. Currently, the most common targets for ICB include the PD-1 receptor and its ligands PD-L1/L2 as well as the CTLA-4 (cytotoxic T-lymphocyte-associated protein 4 receptor and its ligands CD80/86 (5, 6).

Although a large number of new therapeutic options like AA or immunomodulatory agents have been successfully introduced in recent years, most patients develop adaptive or intrinsic resistance mechanisms associated with disease progression. Thus, new even more effective treatment strategies for preventing or overcoming resistance are urgently needed. An overview of current treatment recommendations concerning clinically approved AA and ICB agents according to the European Association of Urology (EAU) in mRCC is given in Figure 1.

## General Composition of the Tumor Microenvironment

The tumor microenvironment (TME) is a highly heterogeneous and dynamic network consisting of several cellular and extracellular matrix components. In this network the heterotypic cellular interactions of myofibroblasts, fibroblasts, neuroendocrine cells, adipose cells, immune-inflammatory cells as well as endothelial cells determine the outcome of tumor progression by promoting tumor growth, tumor dormancy, tumor invasion as well as metastatic growth [reviewed in (7)]. More specifically, tumor cells act either directly by releasing certain factors (e.g., growth factors, cytokines) or indirectly by inducing hypoxia or necrosis, hence modifying the TME by attracting or activating non-tumoral cells like blood and lymphatic endothelial cells, pericytes, carcinoma-associated fibroblasts (CAFs), bone marrow-derived cells or immune, and inflammatory cells. Moreover, tumor cells are able to modify the extracellular matrix, leading mostly to tumor progression. On the other hand, tumor microenvironmental events promote tumor progression by stimulating tumor growth and survival associated with metastasis formation [reviewed in (8)]. Thus, in several tumors like non-small cell lung cancer (NSCLC) or melanoma, but also in RCC, targeting the TME to encapsulate or destroy cancer cells in their local environment has become an attractive novel treatment option with the result that various preclinical and clinical studies are attempting to either stimulate or combat components of the TME. Recently, new techniques such as single-cell transcriptomic sequencing have allowed for in-depth characterization and cataloging of the TME in several tumor types (9–11). For example, Lambrechts et al. identified subtypes in early stage NSCLC 52 stromal cell and even described new stromal cell types, concluding that TME composition is more heterogeneous and pleitropic than primarily appreciated.

## Composition and Impact of the RCC Tumor Microenvironment

As mentioned, RCC is an immunogenic and pro-angiogenic cancer, for which AA therapeutics and ICB are the current mainstay of treatment in the metastatic stage of the disease (Figure 1). In the past years several studies have addressed the characterization of various cell populations within the RCC TME describing a link between the components of the TME and patient outcome as well as therapy response and showing that the TME has become an attractive new target in mRCC treatment. In general, angiogenesis is a physiological process that supplies oxygen and nutrients to the organs, thereby maintaining a stable balance between pro-angiogenic and AA factors. Indeed, tight modifications within this balance like an increase in pro-angiogenic factors can inflame it to pathological angiogenesis associated with the development or progression of cancer by contributing to tumor growth and metastasis formation. Usually, overproduction of pro-angiogenic chemokines like VEGF or members of pro-angiogenic/pro-inflammatory cytokines entitled ELR^+^CXCL (so named for their N-terminal glutamate, leucine, and arginine tripeptide motif preceding the C-X-C chemokine motif) comprising CXCL1, 2, 3, 5, 6, 7, and 8 result in a disruption of the angiogenic balance. Besides the ligands, their receptors (e.g., VEGFR, CXCR) also expressed at the surface and endothelial and tumor cells are key players in stimulating angiogenesis through activation of the RAS/RAF/MEK/ERK and PI3 Kinase/AKT/mTOR signaling pathways, which leads to increased cell proliferation as well as to the production of further angiogenic and inflammatory cytokines.

Already almost 15 years ago, immunotherapy with interferon alfa (IFNα) or interleukin-2 (IL-2) was the pioneer of systemic treatment of RCC. Today, immunotherapy is still an important treatment option for mRCC patients (Figure 1) [reviewed in (12)]. Basically, tumor antigens are presented by dendritic cells (antigen-presenting cells) followed by the modulation of T cell activity by “immune checkpoints” on the surface of T cells, the blockade or stimulation of which consequently results in increased T cell activity—a key step in modulating the anti-tumor immune response (5).

In the following section we will discuss individual key components of the TME in RCC that have implications for disease progression as therapeutic targets or have an impact on biological function:

1. Vascular TME compartments
  - Endothelial cells
    Endothelial cells form the barrier between circulating blood or lymph in the lumen and the rest of the vessel wall. Those endothelial cells that are in constant contact with blood cells are called “vascular endothelial cells,” whereas those in direct contact with lymph are known as “lymphatic endothelial cells.” Endothelial cells are highly metabolically active and play a critical role in many physiological processes like the trafficking of blood cells between blood and underlying tissue, the maintenance of blood fluidity or in innate and adaptive immunity [reviewed in (13)]. However, endothelial cells have also been reported to be involved in diverse pathological processes including tumor initiation and progression, predominantly by contributing to pathological angiogenesis (14).
    Recruitment of vascular endothelial cells from the TME to forward angiogenesis has been demonstrated to play an essential role in the progression of RCC [reviewed in (6, 15, 16)]. On the other hand, endothelial cells can be used as a predictive marker for monitoring therapy as mature circulating endothelial cells (CECs) as well as endothelial progenitor populations (CEPs) reflect the activity of AA agents on tumor neovasculature (17, 18). For example, Farace et al. reported in a patient cohort of 55 mRCC patients previously treated with AA agents (sunitinib or sorafenib), that higher levels of circulating CD45^dim^ CD34^+^VEGFR2^+^ progenitor cells correlate with a poorer outcome, speculating that they might be usable to predict the outcome of AA therapy (19). Similarly, Vroling et al. investigated the behavior of CECs in parallel with hematopoietic progenitor cells (HPCs) in the blood of RCC patients during AA therapy. They analyzed the kinetics of a specific population of small VEGFR2-expressing CECs (CD45^neg^/CD34^bright^), HPCs (CD45^dim^/CD34^bright^) and monocytes in the blood of 24 RCC patients receiving sunitinib using four-color flow cytometry (FCM). Interestingly, CECs (CD45^neg^/CD34^bright^) were increased in RCC patients during treatment with sunitinib. The authors of this study speculated that the increased number of CECs might reflect endothelial cells that became detached or were shed from sunitinib-targeted immature tumorous blood vessels (20). However, the question whether the observed changes in CECs or other circulating subsets of cells are merely a pharmacodynamic marker of sunitinib activity or might have a predictive value remains unanswered in this study and requires further investigation. Recently, a prospective multicenter study in 75 patients assessed the association of CECs with long-term benefit from first-line treatment in ccRCC (SOGUG-CEC-2011-01 study). Interestingly, patients with baseline CECs above the median showed progression-free survival (PFS) (*p* = 0.016) that was significantly longer than those with low CECs (22.2 vs. 12.2 months) (21). Furthermore, CEP/CECs appear to play an important role in AA therapy resistance, as our own data shows that CEP/CEC populations are increased in AA- (sunitinib) treated mRCC patients who become resistant to the drug (22). When reviewing these findings, it is seen that AA therapy induces a more normalized vasculature (decrease in CEP/CEC). On the other hand, at the time of therapy resistance an increase in CEP/CEC levels might represent a more torturous vascular network. Further studies of CEP/CEC dynamics will clarify the impact.
    Concerning the response to immunotherapy, the latest data from our institution including mRCC patients treated with the PD-1 inhibitor nivolumab served to investigate the role of IDO-1 expression in tumor endothelial cells as a predictor of therapy response to the drug. That study showed that IDO-1 overexpression (>10%) was present more frequently in therapy responders than in non-responders, resulting in better PFS during immunotherapy (23). In addition, a recent study assessed biomarkers for either AA, ICB, or a combination of the two and revealed that patients who respond well to AA exert a so-called AA signature characterized by a higher vascular density (high CD31 expression). In contrast, the subgroup of patients with a strong expression of the T-effector (T_eff_) gene signature (T_eff_ High) was positively associated with PD-L1 expression on immune cells and CD8 T-cell infiltration of the T-effector (T_eff_) gene signature (T_eff_ High), being indicative of pre-existing adaptive antitumor immunity (24). In addition, an increase in PFS and ORR was observed in patients with T_eff_ High treated with the combination of ICB (atezolizumab) and AA (bevacicumab).
    Recent evidence suggests that tumor endothelial cells (TECs) differ from normal endothelial cells (11). TECs isolated from RCC patients have been shown to have cytogenetic abnormalities reflecting a classical hallmark of cancer: Akino et al. investigated for the first time chromosomal aberrations in freshly isolated TECs from RCCs and analyzed cell-cell fusion as well as the relationship between progenitor marker-positive cells and TEC aneuploidy in cross-species tumor models. Remarkably, they found that 33% of TECs were aneuploid, while normal endothelial cells were diploid. CD133^+^ (marker for progenitor cells) TECs showed aneuploidy more frequently than CD133^−^ TECs did (25). This finding is highly interesting as TECs have always been assumed to be very homogeneous and not capable of proliferation. However, we now have evidence that TECs show cytogenetic abnormalities and a hyperactivated phenotype (hyper-glycolytic and proliferative). This discovery has important implications because drug resistance will compromise the effectiveness of AA therapies and thus raise the critical issue that stromal cells in TME may also be genetically/morphologically abnormal. This would offer an additional target for cancer therapy and question our general approach to drug development.
    Further important players in cancer development and progression are hormone receptors like the androgen receptor (AR) that is expressed not only in prostate cancer and many other tumors, but also in non-cancerous cell types (26). For example, it has been shown that AR may be used as a prognostic marker to promote RCC progression via increased endothelial cell proliferation and altered HIF-2α/VEGF signaling as AR increases endothelial cell proliferation by modulating the AKT- NF-κB- CXCL5 signaling (27). Moreover, there is evidence that estrogen receptor β (ERβ) could play a promoting role in RCC progression and that targeting the ERβ/TGF-β1/SMAD3 pathway with anti-estrogen ICI182,780 (Faslodex) or with a selective ERβ antagonist 4-[2-phenyl-5,7 bis(trifluoromethyl)pyrazolo[1,5-a]pyrimidin-3-yl]phenol can significantly reduce RCC tumor growth and invasion (28).
  - Lymphatic networks
    The lymphatic system is a network of lymphatic vessels primarily involved in inflammation processes, in fluid and lipid transport as well as in tissue homeostasis [reviewed in (29)]. Like blood, vascular endothelial cells as well as lymphatic endothelial cells play important roles in the trafficking of immune cells, in controlling the microenvironment and in modulating the immune response. Generally, the lymphatic microvasculature is uniquely adapted to continuously remove interstitial fluid and proteins and is an important port of entry for leukocytes and tumor cells [reviewed in (29)]. VEGF-C and VEGF-D are known to play a crucial role in lymphangiogenesis via activation of VEGFR-3 expressed mainly by lymphatic endothelial cells in normal adult tissues. However, it has also been proven that increased lymphangiogenesis is a hallmark of many cancers as the lymphatic system is involved in tumor cell dissemination and consequently distant metastatic growth. VEGF-C is currently the best characterized lymphangiogenic factor acting via VEGFR-3, whose activation is responsible for lymphatic endothelial cell proliferation, migration, and survival. However, VEGFR-3 is also expressed on angiogenic blood vessels, thereby correlating with accelerated tumor progression and/or an unfavorable clinical outcome (30). For example, VEGF-C overexpression in breast cancers has been shown to correlate with lymphangiogenesis and metastasis (31). In preclinical RCC models, endothelial cells chronically exposed to an anti-VEGF antibody proliferate in response to VEGF-C stimulation, whereas naïve endothelial cells are unable to proliferate (32). From the clinical point of view, Dufies and colleagues hypothesized that lymphatic networks driven by VEGF-C may be predictive for therapy resistance in mRCC patients undergoing AA therapy with sunitinib. Indeed, their data confirm that sunitinib stimulates VEGF-C gene transcription and increases VEGF-C mRNA half-life. Moreover, sunitinib stimulated a VEGF-C-dependent development of lymphatic vessels in experimental tumors. Concluding, the authors of that study speculate that destroying tumor blood vessels with VEGF-targeting therapies may induce the development of lymphatic vessels, which could contribute to treatment failure (33).
    However, besides lymphatic networks, other factors like tumor-infiltrating lymphocytes (TILs) are also essential for recruitment of immune cells as the presence of TILs correlates with improved prognosis and therapy response to immunotherapy in several tumor types (34).
  - Mast cells
    In addition to the modulation of the immune response, mast cells are important mediators of angiogenesis. Moreover, mast cells in the TME might function through various cytokines/chemokines (35). In RCC it has been demonstrated that recruitment of mast cells increases angiogenic behavior by modulating PI3K-AKT-GSK3β-AM signaling and that specific inhibitors are able to decrease their recruitment, thereby providing evidence that targeting this pathway is a promising new treatment strategy (36). Interestingly, the number of mast cells in the renal peritumoral zone of 54 RCC patients inversely correlated with 5-year survival, assuming that mast cells might be used as a prognostic marker (37). Similarly, tumor-infiltrating mast cells were also identified from another group as being a powerful, independent prognostic factor for both cancer-specific and radiography-free survival in 188 patients with localized RCC (38). However, a different study showing no significant association between the number of mast cells in tumoral tissue and micro-vessel density was also found (39).
2. Immune cell TME compartments
  Broadly speaking, subdivided macrophages and T cells are the main immune cells of the TME/tumor stroma in RCC. Last year an RCC-specific immune atlas using mass cytometry in-depth immune profiling of samples from 73 RCC patients (no previous systemic therapy) was published. It stated that 17 tumor-associated macrophage phenotypes (TAM), 22 T cell phenotypes as well as a distinct immune composition correlated with patients' PFS defined as the number of days from diagnosis until the first locoregional recurrence, distant recurrence or death. In addition, potential prognostic biomarkers were identified as, for example, a high CA-2 score (high frequencies of either M-11 or M-13 and low frequencies of M-5 macrophages) was associated with a worse clinical outcome. These macrophage subtypes are thus proposed as new targets for RCC treatment (40). In accordance with these findings, still larger data from the RCC Genome Atlas recently described immune gene signatures associated with decreased survival rates, including signatures representing T cells, B cells, macrophages, dendritic cells, and NK cells (41). Most importantly, the authors of this study determined that BAP1, PBRM1 as well as metabolic pathway changes correlate with RCC subtype-specific survival. In addition, DNA hypermethylation/CDKN2A alterations were associated with poor survival in all RCC subtypes. Moreover, an increased Th2 gene signature was identified in each RCC subtype (41).
  - T Cells
    In 2015 Giraldo and colleagues investigated both the infiltration and the localization of CD8^+^ T cells and mature dendritic cells as well as the expression of immune checkpoints (PD-1, LAG-3, PD-L1, and PD-L2) in relation to prognosis by immunohistochemical and immunofluorescence staining using formalin-fixed paraffin-embedded (FFPE) tissue samples from 135 RCC patients. They identified two different patient cohorts, namely one group characterized by a strong expression of immune checkpoints in the absence of fully functional mature dendritic cells associated with poor risk. The second group was characterized by a weak expression of immune checkpoints and mature dendritic cells in peritumoral immune aggregates associated with good prognosis (42). Similarly, the same group analyzed via FACS analysis T cells derived from tumor tissue (TIL), adjacent non-malignant renal tissue (RIL) as well as from peripheral blood lymphocytes (PBL) in a cohort of 40 RCC patients. Remarkably, they identified three different immune profiles, namely (i) immune-regulated (CD8^+^PD-1^+^Tim-3^+^Lag-3^+^ TILs and CD4^+^ICOS^+^ cells with a regulatory T (Treg) cell phenotype) (ii) immune-activated (enriched with oligoclonal/cytotoxic CD8^+^PD-1^+^Tim-3^+^ TILs) as well as (iii) immune-silent (enriched with TILs exhibiting RIL-like phenotype). Of note, only immune-regulated tumors displayed aggressive histologic features as well as a high risk of disease progression in the year following nephrectomy (43).
    Using an immunofluorescence technique, Granier et al. quantified intratumoral CD8^+^ T cells co-expressing the inhibitory receptors PD-1 and Tim-3 in 87 RCC patients. Data revealed that the percentage of tumor-infiltrating CD8^+^T cells co-expressing PD-1 and Tim-3 correlated with an aggressive phenotype and a larger tumor size at diagnosis (44). CD25 is expressed at high levels on Tregs and was initially proposed as a target for cancer immunotherapy. Interestingly, it has been proven that Fc-optimized anti-CD25 depletes tumor-infiltrating regulatory T cells and synergizes with PD-1 blockade in treating several cancer entities including RCC *in vitro*, in mice as well as in patients (45).
    The microenvironment cell population counter (MCP counter) is a new methodology based on transcriptomic markers assessing the proportion of several immune and stromal cell populations in the TME from transcriptomic data using a gene signature for eight immune cell types as well as fibroblasts and vessels (46, 47). In addition, this work group published two papers in which they aimed to stratify mRCC patients into molecular subtypes using MCP counter analyses. Briefly, the data clearly identified four molecular subtypes depending on their TME composition (immune infiltration, MHC1 expression, T- and NK lymphocytes, fibroblastic infiltration) associated with a different prognostic outcome as well as depending on therapy response to the TKI sunitinib (48, 49).
    In July 2018 Wang et al. presented a new empirical approach to dissecting the tumor cells and the TME in RCC using tumorgraft (PDX) RNA-seq data from >1,000 RCC patients. In addition to the identification of 610 novel immune/stromal transcripts, the authors describe a new RCC subtype, namely the inflamed pan-RCC subtype (IS) using DisHet, a three-component dissection algorithm that is able to obtain an empirically defined TME expression signature. Data revealed that IS RCCs are enriched for Tregs, NK cells, neutrophils, macrophages, B-cells, TH1 cells, and CD8^+^ T cells and are importantly associated with poor prognosis and poor survival (50).
  - Tumor-associated macrophages (TAMs)
    TAMs derived from blood monocytes have served as a paradigm for leukocytes and inflammatory mediators in the tumor context. Furthermore, they play a dominant role in cancer-related inflammation. Moreover, TAMs directly stimulate tumor cell proliferation and promote angiogenesis. In addition, they are involved in immune escape processes by producing immunosuppressive cytokines and facilitating tumor dissemination by producing extracellular matrix remodeling enzymes [reviewed in (51)].
    Concerning RCC, in 2009 it was reported for the first time that the presence and the amount of TAMs may correlate with disease prognosis and that strong infiltration of CD163^+^ cells (marker for TAMs) was significantly associated with poor clinical prognosis in an univariate but not a multivariate analysis (52). Similarly, Cros et al. found that the presence of TAMs was associated with a poor prognosis and an early relapse in RCC patients (53). TAMs isolated from RCC produce large amounts of immunosuppressive interleukin-10 (IL-10) and CCL-2, which attracts monocytes to the tumor site. In addition, TAMs harbor enhanced 15-lipoxygenase-2 (15-LOX2) activity resulting in inflammation, immunosuppression, and malignant progression (54). Recently, Motoshima et al. showed that TAMs in metastatic lesions have a greater M1/inflammatory function than those in primary lesions (55). Another highly important feature of TAMs is the induction of angiogenesis as it has been shown in 51 RCC patients that high CD68^+^ TAM density correlates with high microvessel density (56). These data are supported by an additional study demonstrating that VEGFR-1 knockdown leads to reduced macrophage infiltration in the tumor (57). Moreover, another potential molecular mechanism responsible for polarizing monocytes toward a pro-tumoral phenotype has been identified as monocytes from RCC patients displaying a tumor-promoting transcriptional profile exerting functions like angiogenesis and invasion. In addition, a crucial contribution of the IL-1-IL-1R pathway in shaping the tumor-promoting phenotype of these cells was described in that study (58). The same tumor-promoting function and gene profile was mirrored in TAMs isolated from RCC patients and human RCC xenograft tumors (58).
    Based on these TAM characteristics, they are discussed as a target for cancer therapy in RCC in that TAM recruitment would be suppressed with the aim of depleting their number or switching M2 (production of anti-inflammatory cytokines) TAMs to the antitumor M1 phenotype (production of inflammatory cytokines) (51).
  - Cancer-associated fibroblasts (CAFs)
    Cancer-associated fibroblasts (CAFs) are an additional cell type within the TME and trigger tumorigenic features by initiating the remodeling of the extracellular matrix or by secreting cytokines. Fundamentally, CAFs are a subpopulation of fibroblasts with a myofibroblastic phenotype predominantly expressed in cancerous wounds. The most important functions of CAFs are to stimulate angiogenesis and metastatic growth by secreting growth factors such as VEGF, platelet-derived growth factor (PDGF), transforming growth factor-β (TGF-β), platelet epidermal growth factor (EGF) or fibroblast growth factor (FGF). Moreover, secretion of IL-1β by immune cells has been demonstrated to be an initiator of nuclear factor-κB signaling in fibroblasts [reviewed in (52)]. In diverse tumor entities, including prostate cancer, there is evidence that CAFs promote cancer carcinogenesis, cell proliferation and invasion (59). Concerning RCC CAFs have been described as being less abundant, but still present, than other tumor entities like prostate, pancreas or colon are (60). Interestingly, an *in vitro* model studying the interaction of RCC cell lines with CAFs elucidated that co-culture of RCC cells with CAFs increases cell proliferation activity as well as the migration potential as compared with serum-free medium controls promoting CAFs as an innovative TME target for future RCC therapies (61). In line with these data a clinical publication demonstrating that the staining intensity of stromal fibroblasts associated with cancer cells correlates with large tumor diameter (≥4 cm), high-grade (G3/4) tumors, and high-stage (≥pT3) tumors exists. Notably, fibroblast activation protein–positive cases had a significantly shorter survival after five, 10, and 15 years of follow-up (HR 0.31) (62).
3. Influence of tumor cells on the surrounding environment
  Although the topic of tumor cells is beyond the scope of this review, it is important to mention that tumor cells themselves are also able to influence the TME and thus promote tumor growth and metastatic formation mainly by (1) tumor cell-mediated effects on the stromal microenvironment via the release of cytokines/growth factors, (2) tumor cell and stromal fibroblast-driven inflammation and its influence on recruitment and function of immune cells in the TME, or (3) cell-cell and cell-matrix signaling driving tumor cell survival, proliferation, motility, and invasion [reviewed in (63, 64)].

## Rationale and Clinical Implication for Combination Therapies

**Figure 2:**
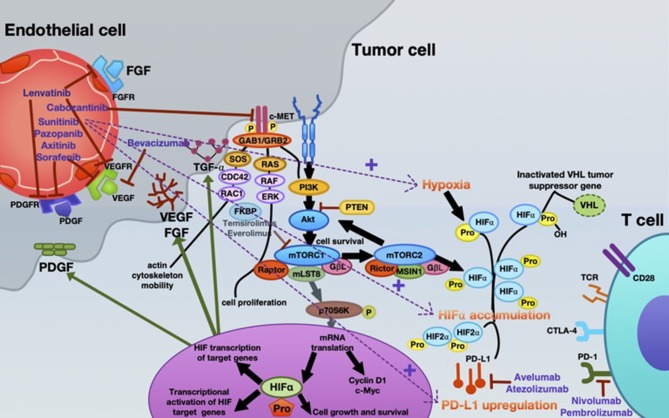
Mechanistic interactions between VEGF-targeted anti-angiogenic therapy and checkpoint inhibitors in the systemic treatment of mRCC. Various VEGF-targeted and mTOR-targeted anti-angiogenic agents are currently approved in mRCC, inhibiting VEGF-A (bevacizumab); VEGFR1-3, FGFR1-4, PDGFR- α, c-kit, and RET-oncogene (lenvatinib); c-MET, AXL and RET, and VEGFR2 (cabozantinib); c-KIT, FGFR, PDGFR, and VEGFR (pazopanib); VEGFR1-3, c-KIT, and PDGFR (axitinib); VEGFR, PDGFR, and Raf family kinases (sorafenib); VEGFRs, PDGFRs, and RET (sunitinib). Everolimus and temsirolimus are specific inhibitors of mTOR and interfere with the synthesis of proteins that regulate proliferation, growth and survival of tumor cells. These agents are more selective for the mTORC1 protein complex, with very little impact on the mTORC2 complex. Nevertheless, increased tumor hypoxia during this anti-angiogenic therapy is the key player for developing TKI resistance, with consecutive HIF-α accumulation. Under hypoxia, PD-L1 upregulation was dependent on HIF-2α in RCC, being associated with simultaneous VEGF overexpression. Moreover, specimens from patients treated with anti-angiogenic therapy were associated with enhanced expression of PD-L1 (1). Thus, combined blockade of PD-L1 (avelumab or atezolizumab) or PD-1 (nivolumab or pembrolizumab) along with inhibition of the angiogenesis pathway is an innovative therapeutic concept in mRCC.

Inhibition of angiogenesis can delay tumor growth, but on the other hand it can also promote metastasis through the existence of abnormal tumor vessels as blood vessel tortuosity in tumors impeded homing of immune cells [reviewed in (65)]. A possible concept for overcoming this confounder is to normalize the tumor vasculature by restoring a balance of pro-angiogenic and AA factors, thereby inducing a more hostile microenvironment and actively stimulating immune activation (6, 66, 67). Recently, new data on vessel normalization demonstrated that type 1 T helper (Th1) cells play a crucial role in vessel normalization (68). As mentioned, both ICB and AA are implicated in the current standard therapy algorithm of mRCC patients. However, recent findings suggest that, ideally, combining either AA agents with ICB or dual ICB may further enhance treatment response. Already in 2008, it was shown that the AA agent sunitinib reverses type-1 immune suppression and decreases Treg in RCC patients (69). In addition, atezolizumab (anti-PD-L1) in combination with bevacizumab (anti-VEGF antibody) enhances antigen-specific T-cell migration (CD8^+^ T cells) in mRCC (70). A schematic overview of the mode of action of AA agents, immunotherapy, and their reciprocal interaction is illustrated in Figure 2.

Currently, several combos combining ICB and AA therapies are being tested in large clinical studies. An overview of ongoing clinical trials combining AA and ICB is provided in Table 1.

**Table 1:** Ongoing clinical studies combining AA and immunotherapies in RCC.

**Study name**	**AA**	**ICB**	**Phase**	**Primary endpoint**	**Therapy lines**	**NCT number**
IMmotion150	Bevacizumab	Atezolizumab	II	PFS (ITT and PD-L1^+^)	First line	NCT01984242
IMmotion 151	Bevacizumab	Atezolizumab	III	PFS (PD-L1^+^) OS (ITT)	First line	NCT02420821
JAVELIN renal 101	Axitinib	Avelumab	III	PFS (PD-L1^+^) OS (PD-L1^+^)	First line	NCT02684006
JAVELIN renal 100	Axitinib	Avelumab	I	MTD	First line	NCT02493751
200249	Pazopanib	Pembrolizumab	II	Clinical efficacy and safety	First line	NCT02014636
KEYNOTE-426	Axitinib	Pembrolizumab	III	PFS, OS	First line	NCT02853331
CheckMate 9ER	Cabozantinib	Nivolumab	III	PFS (ITT)	First line	NCT03141177
CLEAR	Lenvatinib everolimus	Pembrolizumab	III	PFS	First line	NCT02811861
N.a.	Pembrolizumab axitinib	Pembrolizumab	Ib	Safety, treatment efficacy	First line	NCT02133742
N.a.	Pembrolizumab	Pembrolizumab	Ib/II	MTD, ORR	No standard therapies anymore available	NCT02501096

*Status according to clinicaltrials.gov 10/04/2019 OS, overall survival; PF, progression-free survival; ORR, objective response rate; MTD, maximum tolerated dose; AA, anti-angiogenic therapy; ICB, immune checkpoint blockade; N.a., not assessed; IT, intention to treat; PD-L1, programmed death ligand 1. Atezolizumab monoclonal antibody (mAB) PD-L1, axitinib tyrosine kinase inhibitor (TKI)VEGFR 1-3, PDGFR, c-KIT, bevacizumab mAB VEGF-A, lenvatinib TKI VEGFR 1-3, FGFR, PDGFR, RET, c-KIT, nivolumab mAB PD-1, pazopanib TKI VEGFR 1-3, PDGFR, c-KIT, pembrolizumab mAB PD-1, sunitinib TKI PDGFR, VEGFR 1-3, c-KIT, FLT*.

At the ASCO 2018 meeting new data from the IMmotion 151 study (NCT02420821) combining atezolizumab plus bevacizumab in the first line setting in a phase III study vs. the sunitinib were presented. In that study patients were stratified by PD-L1 status (<1 vs. ≥1% PD-L1 expression on tumor-infiltrating immune cells). Interim analyses presented at the meeting showed longer PFS for atezolizumab plus bevacizumab vs. sunitinib in PD-L1^+^ patients, but also in the total patient population (PFS HR for atezolizumab plus bevacizumab vs. sunitinib 0.74 in PD-L1^+^ patients and 0.83 in the total population) (Motzer et al., ASCO 2018 meeting, Abstract # 578). Nevertheless, the second primary endpoint, namely OS in the intention-to-treat population, was ultimately not reached.

As part of the previously conducted study, a randomized phase II IMmotion 150 study (NCT01984242) assessing atezolizumab alone or combined with bevacizumab vs. sunitinib in 305 patients with treatment-naïve mRCC, a biomarker program performing a transcriptome map of angiogenesis and immune-expression profiles, was initiated, whose data were recently published (24). Briefly, the authors evaluated the association between the components of the tumor TME and clinical outcome. Interestingly, exploratory biomarker analyses indicated three different gene expression signatures in the patient population: (i) those with an angiogenetic profile respond to sunitinib (HR 0.31), (ii) patients with an immunogenic profile (T-effector cells) respond to atezolizumab plus bevacizumab (HR 0.5), and (iii) those patients who do not respond to either AA or ICB (24). Finally, the authors of this study concluded that these molecular profiles suggest that prediction outcomes with anti-VEGF and immunotherapy may be possible and offer mechanistic insights into how blocking VEGF might overcome resistance to ICB.

At the recent ESMO Meeting 2018, preliminary findings on the phase III JAVELIN 101 study (NCT02684006) investigating the combination of avelumab (anti-PD-L1 IgG1 monoclonal antibody) and axitinib (VEGFR TKI) including 886 patients were presented *[Motzer R, LBA6_PR - JAVELIN Renal 101: a randomized, phase 3 study of avelumab* + *axitinib vs. sunitinib as first-line treatment of advanced renal cell carcinoma (aRCC)]*. In terms of the primary endpoint (median follow-up time 9.9 months), the combination of avelumab plus axitinib significantly improved median PFS in the overall population (13.8 vs. 8.4 months, HR 0.69, *p* = 0.0001) regardless of PD-L1 status or risk category. In terms of confirmed objective response, 51% of all patients had an objective response with axitinib plus avelumab, compared with 26% of patients on sunitinib. In the PD-L1-positive population, a similar benefit was observed (55 vs. 26%). The second primary endpoint, OS in the PD-L1^+^ population, has not yet been reached and, thus, OS data are immature after a median follow-up of only 12 months (avelumab plus axitinib) and 11.5 months for sunitinib.

Promising oncological results achieved in the open-label, dose-finding, non-randomized phase Ib study have been demonstrated for the combination of pembrolizumab (anti-PD-L1 IgG1 monoclonal antibody) and axitinib in the first-line setting including 52 treatment-naïve advanced RCC patients. Median PFS was 20.9 months, regardless of PD-L1 status (median PFS PD-L1^+^ vs. PD-L1^−^ 20.7 vs. 22.1 months). The objective response and the complete response rate were astonishing at 73 and 8%, respectively (71). Thus, a phase III trial (KEYNOTE-426) is ongoing and compares axitinib plus pembrolizumab with sunitinib monotherapy (NCT02853331). The latest data from the Genitourinary ASCO meeting held in February 2019, simultaneously published in the *New England Journal of Medicine*, demonstrated that at a median follow-up of 12.8 months combination therapy was associated with a 47% reduction in the risk of death as compared to the comparator sunitinib. In addition, the ORR was 59.3% with the combination vs. 35.7% with sunitinib. Furthermore, the duration of response was longer in patients treated with combination therapy, with the median not yet reached vs. 15.2 months with sunitinib (72).

## Future Treatment Concepts

Although the AA concept is of great medical interest, as it shapes the behavior of the TME components, thus exerting many synergistic effects, to the best of our knowledge no new AA targets are currently under clinical evaluation. However, we feel that in the coming years the better characterization of TECs might lead to promising new target structures.

However, besides the ICB blockade, new concepts of immune stimulation have recently been identified. Nucleic acid sensing plays an essential role in innate immune response induction. For example, RIG-I and STING are key mediators of nucleic acid sensing and thus agonists are in early clinical development.

### STING Agonist–MK-1454

STING (stimulator of interferon genes) is a key mediator of innate immunity. In the past years evidence has emerged to show that the STING pathway is involved in the induction of the anti-tumor immune response, leading to the development of STING agonists as stimulators of STING with immune-activating and anti-neoplastic activities (73). STING agonists bind to STING and activate the STING pathway, promoting IKK-related kinase TANK-binding kinase 1 signaling as well as activating nuclear factor-kappa B and interferon regulatory factor 3 in immune cells in the TME, thereby increasing the production of pro-inflammatory cytokines (74). Currently, a phase I clinical trial, testing the STING agonist MK-1454 as a monotherapy or in combination with ICB in patients with advanced or metastatic solid tumors is under way (NCT03010176) (https://clinicaltrials.gov).

### RIG-I Agonist–MK-46212

The retinoic acid-inducible gene I (RIG-I) is a pattern recognition receptor that plays a key role in recognizing RNA viruses. In general, RIG-I-like receptors are expressed in most tissues, including cancer cells. Recently, it was demonstrated in preclinical studies that cancer cells can be induced to mimic a viral infection using RLR ligands to activate a cytosolic RNA-sensing pathway and interferon (IFN) response (75). This activation can result in the stimulation of cytotoxic immune cells, such as NK and CD8^+^ T cells, thus destroying cancer cells via extrinsic and intrinsic apoptosis (76). Consequently, activation of RIG-I-like receptors, by means of synthetic ligands or oncolytic virus in tumor cells, can induce cell death in an IFN-dependent or IFN-independent manner. To the best of our knowledge two clinical studies are currently evaluating safety and antitumor activity of the RIG I agonist MK-4621 as a monotherapy and in combination with ICB (pembrolizumab) in patients with advanced/metastatic solid tumors (NCT03739138, NCT03065023) (https://clinicaltrials.gov).

## Conclusion

In recent years, targeting the TME rather than exclusively the tumor cells themselves has become a key concept. Preclinical and early clinical data provide a strong hint that especially endothelial cells and immune cells, the key components of the TME, are strongly involved in RCC progression. Thus, numerous studies are currently under way to test the impact of simultaneous inhibition of angiogenesis and immune checkpoints. The first preliminary results are promising, however final outcomes of large phase III studies remain awaited, before final conclusions can be drawn. In addition, new players like STING agonists or RIG I activators have demonstrated promising early results toward overcoming resistance to conventional therapies.

## Author Contributions

IH and AP: literature research, drafting the manuscript, and revision. RP: figures and tables and critical revision of the manuscript.

### Conflict of Interest Statement

IH: speaker fees and travel grants from Astellas, Janssen, Bayer, Sanofi, Ferring and Roche, Pfizer, advisory board for Bayer, research grant from Bayer. AP: speaker's fee, travel grants and fees for advisory board members from Astra Zeneca, Roche, MSD, and Pfizer. RP: advisory boards for Ipsen, Pfizer, Merck, MSD, BMS and Roche; speaker fees and travel grants from BMS, Merck, MSD, Pfizer, Roche and Pierre Fabre.
